# Strategy to Enhance the Collapse Capacity of Composite Cylindrical Tubes: Experiments and Simulations

**DOI:** 10.3390/ma18071458

**Published:** 2025-03-25

**Authors:** Siddharth Jain, Akash Pandey, Arun Shukla

**Affiliations:** Dynamic Photo-Mechanics Laboratory, Department of Mechanical, Industrial and Systems Engineering, University of Rhode Island, Kingston, RI 02881,USA; siddharth.jain@uri.edu (S.J.); akash_pandey@uri.edu (A.P.)

**Keywords:** carbon fiber-reinforced composite, dynamic buckling, implosion, underwater structures, buckling simulations, parametric study

## Abstract

The effects of adding circumferential groove geometries on the collapse capacity of carbon composite cylindrical tubes were investigated experimentally and numerically. Tubular specimens, both with and without grooves, were imploded hydrostatically in a water-filled pressure vessel facility. High-speed imaging captured the collapse behavior, while dynamic pressure transducers recorded the transient collapse pressure history of the implosion event. The results indicate that the collapse capacity is improved by up to 20% by adding a circumferential groove. A numerical model of the hydrostatic buckling was developed using ABAQUS 2022 software and validated against experimental results. A parametric study was conducted by varying the depth, steepness, and number of grooves. The results showed that deeper grooves tended to partition the tubes into sections that collapsed locally at higher pressures. Additionally, reducing the groove steepness increased the collapse capacity up to a certain threshold, beyond which the capacity decreased. Tubes with more grooves collapsed in higher modes and consequently at higher pressures. This provides a tool by which the groove geometry and pitch distance can be adjusted to achieve the desired collapse mode and capacity.

## 1. Introduction

This study numerically explores the enhancement of collapse pressures by the addition of different types of grooves in composite tubes. In the past, researchers have shown great interest in the buckling of shell geometries for deep sea environments [1,2,3,4]. Underwater marine structures, including offshore pipelines [5,6,7] and underwater unmanned vehicles (UUVs) [8,9], are susceptible to implosion caused by geometric instability when subjected to deep sea hydrostatic pressure. As a result, the marine industry has been working to enhance the longevity and performance of these structures and vehicle systems. Composites offer numerous advantages over metallic structures, such as enhanced corrosion resistance and the ability to withstand greater operating depths for underwater applications. These materials are also favored for their low thermal, acoustic, and magnetic signatures, making them attractive to various industries [10,11,12]. The collapse behavior of these structures and the transient pressure produced during their implosion have been extensively studied by many researchers [13,14,15,16,17,18,19,20,21]. The shock-induced implosion phenomenon at subcritical pressures has been experimentally investigated [22,23]. Several studies have demonstrated that buckling failure can vary significantly for composite structures [24,25,26,27,28]. Enhancing the collapse capacity of implodable structures has also been a focus of interest for the research community. Previously, various geometries and spacing configurations of external and internal ring stiffeners have been studied to strengthen implodable structures [29,30,31,32]. Ngwa et al. [33] investigated the buckling behavior of cylinders reinforced with internal ring stiffeners. They observed a significant increase in the critical hydrostatic pressure required to initiate geometric instability, which also depended on the axial positioning of the rings. Researchers have also conducted numerical studies to optimize the geometry and spacing of the ring stiffeners [34,35]. The use of an external or internal ring as a buckle arrestor increases the collapse capacity, though this improvement is accompanied by a notable increase in mass.

In this study, a strategy is presented to enhance the critical collapse pressures of cylindrical composite tubes by modifying their geometry. Specifically, a cylindrical section of the tube was replaced with an anti-clastic curved section (groove section), which increased the tubes’ stiffness. Underwater implosion experiments were conducted, subjecting the composite tubes, both with and without the groove geometry, to hydrostatic pressure loading. High-speed imaging during the experiments was employed to observe and analyze the deformation behavior of both types of tubes. The tubes with grooves consistently collapsed at higher pressures.

A finite element (FE) model was then developed to simulate the hydrostatic loading of the composite tubes and estimate their collapse pressures. The experimental results were used to validate the FE model. Once validated, the FE model was employed in a parametric study to explore the effect of the groove geometry on the collapse pressure enhancement. The study examined how various groove geometries, and the number of grooves, influenced the collapse pressures of tubes with different geometries. The groove geometry was modified by adjusting the groove depth and groove steepness. The parametric study revealed that tubes with grooves of a greater depth experienced localized collapse, leading to higher collapse pressures. Reducing the groove steepness up to a certain point resulted in an increase in collapse pressures, beyond which the collapse pressures began to decrease. Tubes with a higher number of grooves exhibited an increased collapse capacity and collapse at higher modes. The groove geometry and the pitch distance can be adjusted to achieve the desired collapse mode and collapse capacity. These findings highlight a promising design approach for enhancing the collapse resistance of structures subjected to extreme hydrostatic conditions.

## 2. Materials, Experimental Procedures, and Modeling

### 2.1. Specimen Manufacturing and Material Properties

**Figure 1:**
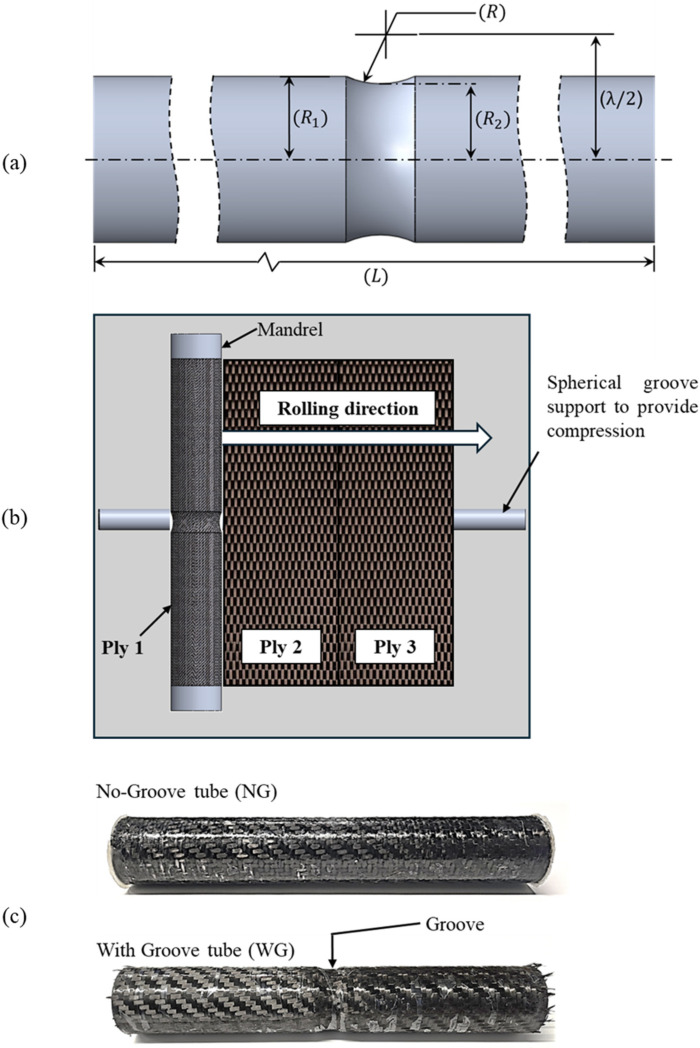
(**a**) Schematic of mandrel for Tubes with-grooves. (**b**) Schematic of roll-wrapping procedure. (**c**) Manufactured tube specimens.

Composite cylindrical tube specimens with a groove in the mid-plane, as well as without a groove, were manufactured in-house using balanced, twill-woven carbon fiber prepregs manufactured by Gurit, Albacete, Spain. The tube specimens were manufactured using a roll-wrapping process, wherein prepregs were roll-wrapped onto an aluminum mandrel. Each specimen consisted of three layers of prepreg, with the fiber orientation maintained in the meridional and hoop directions. Composite tube specimens with no groove were manufactured using a 1 mm thick and 460 mm long hollow aluminum tube of 51 mm outer diameter mandrel. A two-part detachable solid aluminum mandrel was used to fabricate the composite tubes with a groove in the midsection. The mandrel components were symmetric with respect to the plane of partition. A dowel pin and hole arrangement were provided to facilitate the easy assembly and disassembly of the mandrel sections, while ensuring coaxiality. The solid mandrel comprised a 51 mm outer diameter and 460 mm total length at assembly, with a machined circular groove in the midsection of the assembled mandrel. Figure 1a illustrates mandrel geometry with dimension details, including (a) mandrel radius $\left(R_{1}\right)$, (b) mandrel length (L), (c) root radius ($R_{2}$), and (d) a distance parameter (λ/2), which defines the distance between the center of curvature of the groove and the axis of the mandrel. The prepregs wrapped over the mandrel were cured at 120 °C, with a dwell time of 1 h, and shrink tape (make: Composite Envisions, Wausau, WI, USA) was employed for the compression and consolidation of the prepregs. Figure 1b presents a schematic of the manufacturing process. The manufacturing process creates a replica of the mandrel features on the inner surface of the composite tube specimens. The geometric details of both specimens are delineated in Table 1.

A thin coat of two-part polyaspartic polyurea resin MPC-290 (make: MPC Coatings, Laval, QC, Canada) was applied on all the manufactured tubes to ensure leak-proofing. Figure 1c shows photographs of the manufactured tubes.

The mechanical properties characterization of the prepreg material was carried out in accordance with ASTM standard procedure, and the results are provided in Table 2.

### 2.2. Experimental Setup

**Figure 2:**
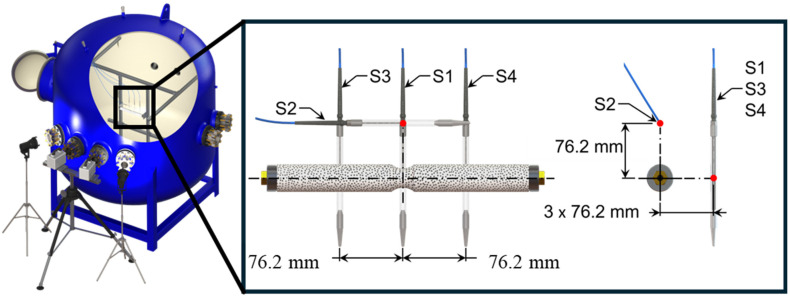
Implosion facility and specimen fixture assembly, along with the dynamic pressure sensor arrangement.

Implosion experiments were carried out at the University of Rhode Island’s Dynamic Photomechanics Laboratory (DPML) using a hydrostatic implosion pressure vessel facility. The facility features a cylindrical pressure vessel with a 2 m diameter and ellipsoidal top and bottom ends. To facilitate imaging and specimen illumination, optically clear windows are positioned around the vessel’s equatorial plane. The specimens inside the vessel can be subjected to hydrostatic pressures up to 6.90 MPa. Figure 2 illustrates an implosion specimen positioned within the implosion facility, along with the dynamic pressure sensor arrangement.

The composite tube specimens, secured at both ends using aluminum end caps, were firmly mounted on an aluminum frame inside the vessel. The end caps, each equipped with O-ring seals, ensured leakproof tubing. Each end cap extended 12.7 mm into the shell on both sides, maintaining an unsupported shell length of 305 mm, with simply supported boundary conditions at the ends. Additionally, a two-part, epoxy-based adhesive (Double/Bubble, Hardman, NJ, USA) was applied at the interface between the end caps and the machined shell to ensure redundancy in watertight integrity.

Four dynamic pressure sensors (make: PCB Piezotronics, Depew, NY, USA; model: W138A05) were positioned around the specimen to capture the transient pressure histories generated during the implosion event. The pressure sensor arrangement is illustrated in Figure 2. The axially offset sensors were strategically placed to ensure that, in the case of a localized collapse where either the left or right partition of the cylinder buckled first, a sensor would be positioned directly at the midplane of each subregion. The output from the pressure sensors was transmitted through a signal conditioner (model: 428C, PCB Piezotronics) and subsequently recorded using a digital oscilloscope (model: DPO 3034, Tektronix, Beaverton, OR, USA). The signal output from the digital oscilloscope was fed to the high-speed cameras to capture the deformation behavior of the composite tubes. Two Photron FASTCAM SA1.1 cameras (Photron, San Diego, CA, USA) recorded stereo images of the event at a rate of 30,000 frames per second.

To initiate the hydrostatic loading of the tubes, the pressure vessel was filled with water, leaving a small air pocket at the top. Compressed nitrogen was introduced into this air pocket, increasing the pressure in both the air and the water at a rate of 6.90 kPa/s until the shell buckled.

### 2.3. Numerical Modeling

**Figure 3:**
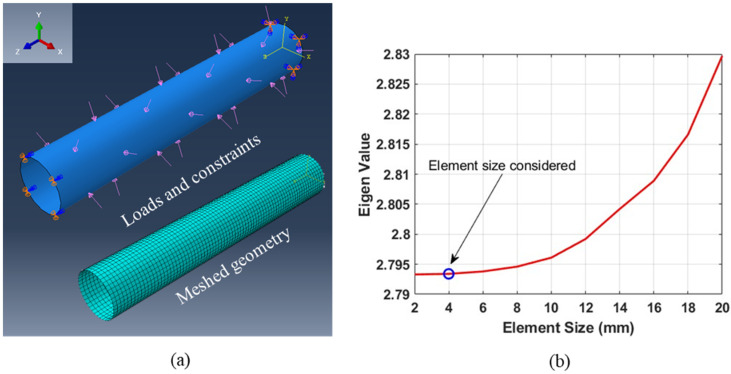
FE model: (**a**) Boundary, loading conditions, and meshed geometry. (**b**) Mesh convergence results indicating the element size considered.

A finite element method-based numerical model was developed to evaluate the effect of various geometric parameters of the groove on the collapse behavior of the composite tubes. The numerical simulations were carried out using Abaqus/CAE 2022 software from Dassault Systèmes Simulia Corp., Johnston, RI, USA. The composite tube specimens were modeled as a 3D deformable shell geometry, with the composite material layup defined such that the principal directions of the material were along the meridional and hoop direction. The elastic constants evaluated for the twill-woven carbon fiber prepregs, as detailed in Table 1, were used to define the material properties. The thickness of each ply was modeled as 0.55 mm, based on the thickness measurements on the manufactured tube specimens. The boundary conditions were applied at both ends of the tube. All the translational displacements (U1, U2, U3) were arrested at one end of the tube, with only axial displacement (U3) allowed at the other end to replicate the experimental conditions. A uniform unit pressure load was applied to the external surface of the shell geometry to simulate hydrostatic pressure. The shell geometry was modeled using quadratic quadrilateral shell elements (S8R). A mesh convergence study was conducted for the first buckling eigenvalue, and based on the results, the shell geometry was discretized with an element size of 4 × 4 $mm^{2}$. For all the tubes with grooves, the groove region was discretized with 10 elements along the meridional direction. Figure 3a illustrates the cylindrical shell model with boundary and loading conditions, while Figure 3b presents the results of the mesh convergence study. A linear perturbation buckling analysis [36] procedure was employed to estimate the critical pressure for the onset of geometric instability in the composite tubes. The simulations were limited to the collapse of the cylinders and did not explore the fracture behavior of the tubes and the fluid response in the post-buckling phase.

## 3. Results and Discussion

Underwater implosion experiments were performed to understand the effect of groove geometry on the collapse capacity of composite tubes. A finite element (FE)-based numerical model, validated through experiments, was used to conduct a parametric study. The study aimed to investigate the influence of groove geometric parameters on the collapse capacity of composite cylindrical tubes. The following subsections discuss the experimental results, followed by the parametric study results, with an in-depth analysis of the effect of the geometric parameters on cylindrical tubes of different configurations.

### 3.1. Experimental Results

The experimental results, comprising the collapse behavior of the composite tubes, the transient pressure associated with the implosion event, and the post-experiment visual observations on the specimens, are discussed in the following subsections. Further, the experiment results highlighting the critical pressure are summarized in Table 3.

#### 3.1.1. Deformation Behavior

The hydrostatic pressure differential across the wall of a composite tube, upon reaching a critical magnitude $(P_{collapse}$), leads to geometric instability in the structure. Consequently, the structural walls buckle inwards. The deformation behavior of the composite tubes during the collapse phase was captured using high-speed imaging. Both the composite cylindrical tube specimens and composite tubes with a groove in the midsection exhibited global failure.

**Figure 4:**
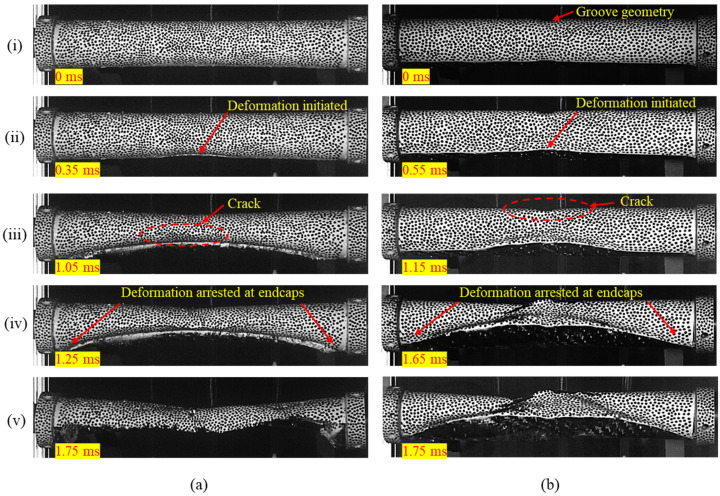
Chronology of events during implosion (**i**–**v**) for (**a**) no-groove tube and (**b**) tube with groove.

Regardless of the specimen configuration, all composite tubes exhibited Mode II buckling, where the circular cross-section transformed into a two-lobed geometry during collapse. The Mode II collapse here refers to the angular buckling mode, which is discussed in detail by Matos et al. [16]. Figure 4a,b illustrate the sequence of significant events during the collapse phase for the no-groove and grooved configurations, respectively. The photographs corresponding to instance (i) show the undeformed specimens. Geometric instability initiated in the middle portion of the tube, as shown in instance (ii), and further propagated toward the ends. Instance (iii) captures the cracks originating at the midsection in the plane of collapse, occurring as tube wall deformation progressed.

As buckling propagated toward the specimen ends, large deformations developed along the tube length. Due to the inherent brittleness of carbon fiber-reinforced composite material, cracks propagated along the length of the tube specimens, leading to catastrophic fracture failure with multiple cracks, as shown in instances (iv) and (v). Even though the collapse behavior was similar, the critical pressure for composite tubes with a groove was 20% higher than that of tubes without a groove. The collapse pressure magnitudes measured during the experiments are listed in Table 3.

#### 3.1.2. Dynamic Pressure History

**Figure 5:**
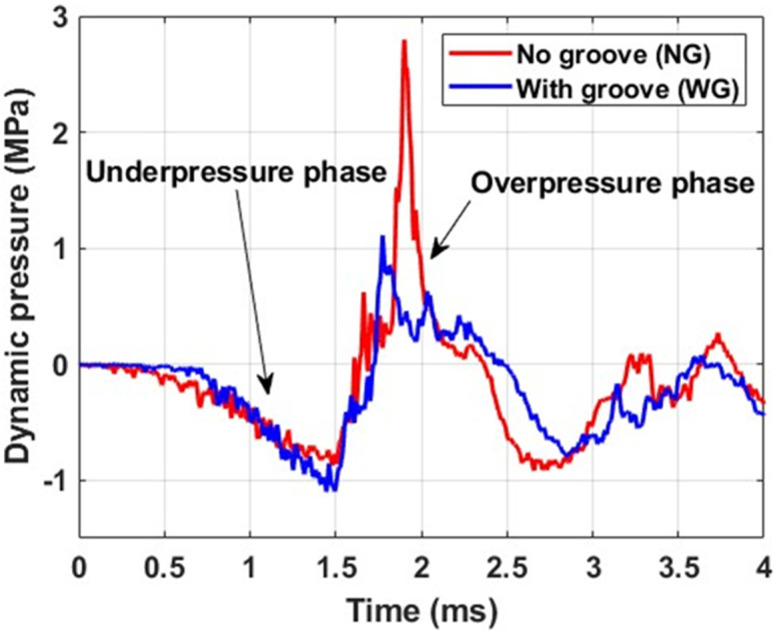
Pressure–time history (typical).

The dynamic pressure variation in the vicinity of the tube, resulting from its implosion, was recorded at the locations specified in Section 2.2. A typical pressure–time history at the sensor 2 location for tubes with and without grooves is presented in Figure 5. Since buckling initiated in the midsection of the tube in all experiments, the dynamic pressure recorded at this location was compared for both specimen configurations.

As the tube walls collapse inward, the surrounding fluid gains momentum, causing a drop in pressure relative to the hydrostatic pressure. This stage is referred to as the underpressure phase. When the collapsing tube walls make contact and buckling propagates along the tube length, the motion of the surrounding fluid comes to an abrupt halt. This results in a high-amplitude pressure surge, defined as the overpressure phase. The dynamic pressure during the underpressure regime was similar for both configurations, as evident in Figure 5. The average specific impulse measured for the duration of the underpressure phase was 555 Pa-s and 530 Pa-s for the composite tubes without grooves and with grooves, respectively. Further, the magnitudes of the peak dynamic overpressure recorded for the tubes with grooves was 55% less than the specimens without groove.

#### 3.1.3. Postmortem Inspection

**Figure 6:**
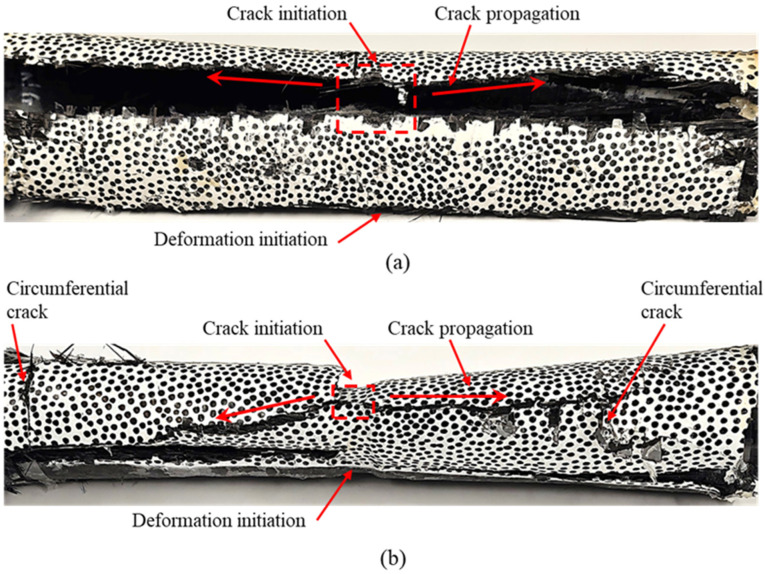
Postmortem images of the specimens: (**a**) No groove (NG). (**b**) With groove (WG).

A post-experiment visual inspection was performed for all the specimens. Figure 6 illustrates the collapsed composite tube specimens. Both the tubes with and without grooves showed similar observations at failure locations. The onset of geometric instability caused the structural walls to buckle inwards. This instability initiated at the middle portion of the tube, resulting in large deformations, as shown in Figure 4. Due to excessive flexural deformations and the inherent brittleness of the carbon fiber-reinforced composite material, cracks were observed in the middle portion of the tube. As the deformation propagated towards the tube ends, these longitudinal cracks also propagated and reached the tube ends, as shown in Figure 6. For tubes without grooves, the longitudinal crack propagated along a straight line, reaching the ends, which resulted in complete wall-to-wall contact and the collapse of the tube. However, for the tubes with grooves, when the crack reached the end, along with wall-to-wall contact, circumferential cracks were also observed.

### 3.2. Numerical Study Results

An FE-based numerical model, validated through experiments, was used to conduct a parametric study. The study aimed to investigate the influence of groove geometric parameters on the collapse capacity of composite cylindrical tubes. The following subsections discuss the validation of the numerical model, followed by the parametric study results, with an in-depth analysis of the effect of geometric parameters on cylindrical tubes of different configurations.

#### 3.2.1. Validation of Numerical Model

A finite element model was used to estimate the critical collapse pressure of the composite tubes under hydrostatic loading. The collapse pressure predictions from the linear buckling analysis results were compared to the average experimental collapse pressures of the tubes with no grooves and with grooves (geometry details provided in Table 1), to validate the FE model. The collapse pressures predicted by the simulations closely match the average experimental values, with a deviation of 1.8% for tubes without grooves and 6% for tubes with grooves. The collapse pressure predicted by FE analysis and the mean collapse pressure obtained experimentally are summarized in Table 4.

**Figure 7:**
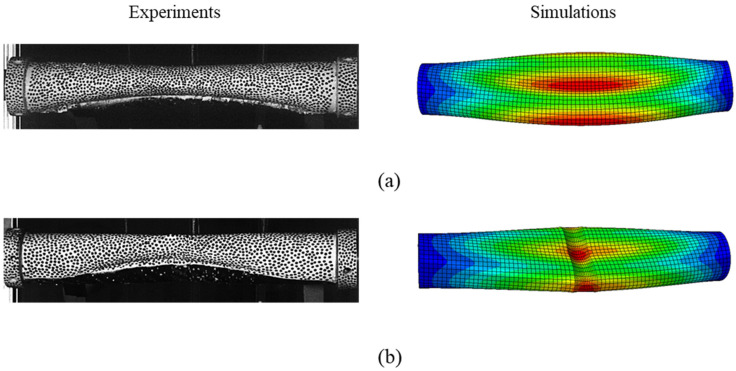
FE model validation illustrating the deformation behavior of the tubes: (**a**) No groove (NG). (**b**) With groove (WG).

The collapse mode of the tube was also very well captured in the linear buckling analysis. Figure 7 illustrates the global collapse behavior of the specimens, both in experiments and simulations. The Mode II global collapse observed in the experiments for the tubes without grooves, as well as with grooves, was also evidenced in the buckling analysis results.

#### 3.2.2. Parametric Study

A parametric study was performed to understand the effect of groove geometry on the collapse pressure of composite tubes. To further understand the efficacy of the grooves, composite tubes of different lengths and wall thicknesses were selected for this study.

The geometry of a baseline composite tube with no groove was defined using three independent parameters: tube length (L), inner radius ($R_{1}$), and shell thickness (t). In all simulations, the inner radius ($R_{1}$) was maintained at 25.4 mm (1 inch). Furthermore, varied configurations of baseline composite tubes were obtained by permutating four different tube lengths and three different shell thicknesses selected for the study. The four tube lengths were defined in proportion to the inner radius of the tube as 8$R_{1}$, 12$R_{1}$, 16$R_{1}$, and 20$R_{1}$. Three different shell thickness values were selected by varying the number of plies (N) among two, three, or four. In all numerical simulations, the material properties from Table 2 were used to define the elastic constants for each ply.

For composite tubes with grooves, the tube geometry was based on the previously defined baseline composite tube with no grooves. The groove geometry in these tubes with grooves was defined using two parameters: root radius ($R_{2}$) and distance parameter (λ/2). The distance parameter (λ/2) defines the distance between the center of curvature of the groove and the axis of the tube. Variants of the groove geometry were obtained by various combinations of three different root radii ($R_{2}$) and five different values of the distance parameter (λ/2) selected for the study.

**Figure 8:**
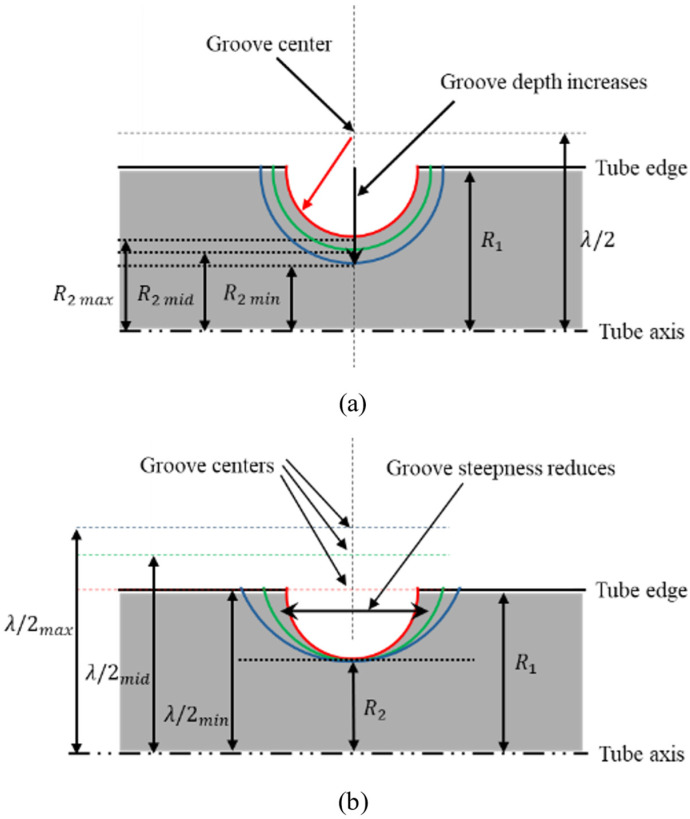
(**a**) Decrease in groove depth with an increase in root radius. (**b**) Decrease in groove steepness with an increase in distance parameter (λ/2).

Three different values of the root radius ($R_{2}$) were defined in proportion to the inner radius of the tube as 3$R_{1}$/4, 7$R_{1}$/8, and 15$R_{1}$/16. The minimum value of $R_{2}$ corresponds to the groove with maximum depth, while the maximum value corresponds to the groove with minimum depth, as shown in Figure 8a. Five different values of the distance parameter (λ/2) selected for the study were defined in proportion to the inner radius as $R_{1}$, 1.25$R_{1}$, 1.5$R_{1}$, 1.75$R_{1}$, and 2$R_{1}$. The minimum value of λ/2 corresponds to the steepest groove, while the maximum value corresponds to the shallowest groove, as shown in Figure 8b.

The effect of the number of groove pitch distances on the collapse pressure of the tubes was also studied by introducing multiple grooves, placed equidistantly along the tube length. The number of grooves (G) was selected as one, two, or three.

Table 5 presents a summary of all the parameters considered in the study. A total of 540 simulations were performed, considering all permutations of tube and groove geometries, to study their effect on collapse pressure.

##### Results from Parametric Study

**Figure 9:**
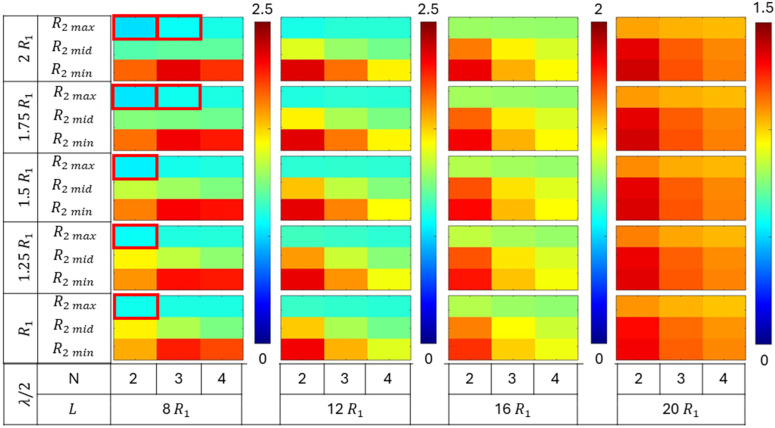
Normalized collapse pressures of tubes with one groove of varying length, thickness, and groove geometry.

**Figure 10:**
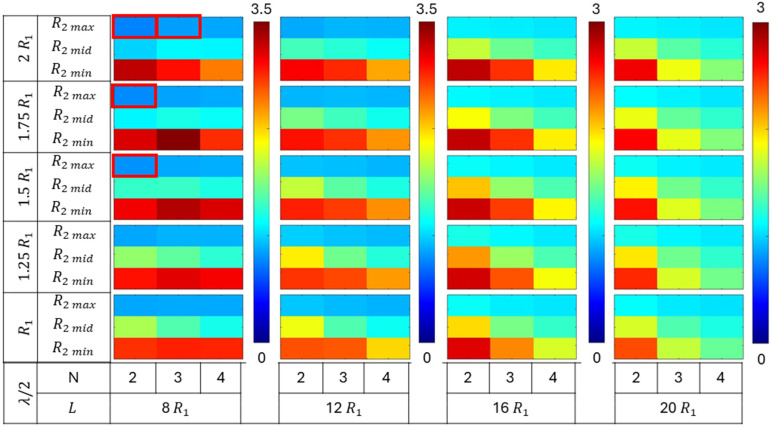
Normalized collapse pressures of tubes with two grooves of varying length, thickness, and groove geometry.

**Figure 11:**
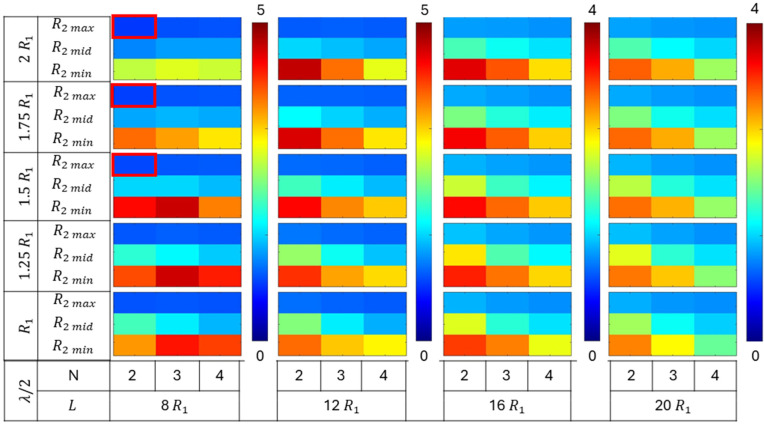
Normalized collapse pressures of tubes with three grooves of varying length, thickness, and groove geometry.

The collapse pressures were evaluated for a total of 540 permutations of various geometric parameters defined earlier, comprising tube length (L), number of plies (N), root radius ($R_{2}$), distance parameter (λ/2), and number of grooves (G). The efficacy of different groove configurations on collapse capacity was compared for tubes with varying geometries. The collapse pressures obtained for the grooved configuration tubes were normalized with respect to the critical pressure of their corresponding no-groove tube geometries. The normalized collapse pressure results were segregated based on the number of grooves in the tube, i.e., G = 1, 2, and 3. Each group comprised 180 simulation results. The normalized collapse pressure for each group is shown in Figure 9, Figure 10 and Figure 11, corresponding to tubes with one, two, and three grooves, respectively.

All three figures (Figure 9, Figure 10 and Figure 11) show the variation in normalized collapse pressure with respect to four geometric parameters. The tube length (L) and the number of plies (N) are represented on the horizontal axis, while the distance parameter (λ/2) and root radius ($R_{2}$) are represented on the vertical axis. Each figure comprises four columns, with each column representing a tube length (L) of $8R_{1}$, $12R_{1}$, $16R_{1}$, or $20R_{1}$. The color bar corresponding to each column represents the range of normalized collapse pressures, with red indicating the maximum and blue indicating the minimum values.

Within each column, the number of plies (N), which governs tube thickness, varies horizontally as N = 2, 3, and 4. On the vertical axis, the data are divided into five groups, with each group comprising three rows. Each group represents a different distance parameter (λ/2) value: $R_{1}$, ${1.25R}_{1}$, $1.5R_{1}$, ${1.75R}_{1}$, and $2R_{1}$. Each row within a group corresponds to a root radius ($R_{2}$) value of ${3R}_{1}/4$, $7R_{1}/8$, or $15R_{1}/16$.

Figure 9 illustrates the normalized collapse pressures for tubes with only one groove at the midsection of the tube. The collapse capacity increased as the root radius ($R_{2}$) decreased. The highest increase was observed in tubes with a minimum root radius ($R_{2\;min}$), followed by those with medium ($R_{2\;mid}$) and maximum ($R_{2\;max}$) root radii. Furthermore, the effect of the root radius ($R_{2}$) was consistent regardless of the number of grooves in the tube, as shown in Figure 10 and Figure 11.

However, increasing the number of grooves (G) further enhanced the collapse capacity of the tubes. The maximum normalized collapse pressures obtained from the simulations were 2.5, 3.5, and 5, for G = 1, 2, and 3, respectively.

The increase in the collapse capacity was maximum for the tubes with a shorter length, and it decreased monotonically with the increase in the tube length (L). Tubes with lengths of $8R_{1}$ and $12R_{1}$ demonstrate a more significant increase in collapse pressures from the addition of a single groove compared to longer tubes, as shown in Figure 9. This indicates that the stiffness provided by a single groove was less significant in longer tubes. However, tubes of all lengths exhibited a notable increase in collapse pressures with the addition of multiple grooves, as shown in Figure 10 and Figure 11. This suggests that a greater increase in collapse pressure occurs with a higher number of grooves per unit length.

An increase in collapse capacity was observed for tubes regardless of the number of plies (N). However, tubes with lengths of $12R_{1}$ or greater exhibited a greater increase in collapse pressures for tubes with two plies, followed by tubes with three and four plies. While, for tubes of a length of $8\;R_{1}$, the greatest increase in collapse pressures was observed for tubes with three plies.

The increase in the distance parameter (λ/2) resulted in an increase in collapse capacity for single-groove tubes with a minimum root radius ($R_{2\;min}$). However, for tubes with a medium root radius ($R_{2\;mid}$), the highest increase in collapse pressures was observed at intermediate values of the distance parameter. These observations are shown in Figure 9. For tubes with multiple grooves, those with lengths of $12R_{1}$ or greater exhibited similar trends to single-groove tubes. However, tubes with a length of $8R_{1}$ displayed a unimodal trend in collapse pressure with variations in the distance parameter. This contrasting trend in collapse capacity, evident in Figure 9, Figure 10 and Figure 11, suggests a strong interaction and competing response between the distance parameter (λ/2) and root radius ($R_{2}$).

The groove configurations that exhibited a negative effect and led to a decrease in the collapse capacity with respect to no-groove tubes are highlighted in “red” in Figure 9, Figure 10 and Figure 11.

Overall, the results from the parametric study suggest that the optimal groove geometry is not unique and varies with the tube geometry. The effect of the geometric parameters root radius and distance parameter are discussed in greater detail in the subsequent subsections.

##### Effect of Root Radius

The effect of the root radius was studied for tubes with one, two, and three grooves across all configurations. The root radius $\left(R_{2}\right)$ was varied to modify the groove depth, with the minimum value of $R_{2}$ corresponding to the maximum groove depth and vice versa, as shown in Figure 8a.

**Figure 12:**
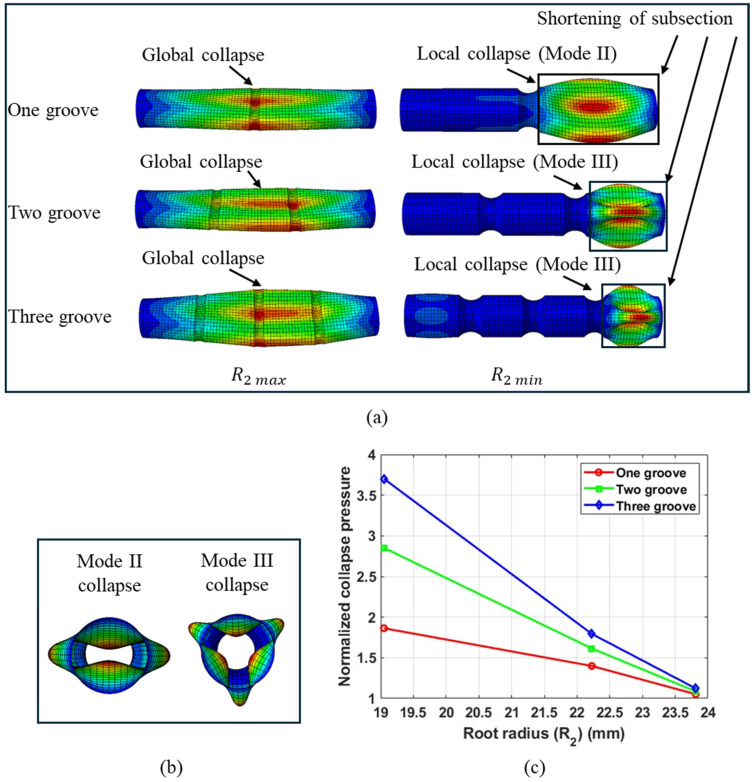
(**a**) Deformation behavior of tubes featuring one, two, and three grooves for maximum and minimum root radii. (**b**) Mode II and Mode III collapse deformed shape. (**c**) The normalized collapse pressure as a function of root radius. Tube length = $12\;R_{1}$, No. of plies = 3, and distance parameter = $1.5\;R_{1}$.

A global trend of increasing collapse pressure with decreasing root radius ($R_{2}$) was observed across the range of grooved tube geometries selected for the parametric study. Upon further examination of the deformed geometry of the tubes, two distinct collapse behaviors were identified. Tubes with the minimum root radius ($R_{2\;min}$) exhibited local collapse, whereas those with the maximum root radius ($R_{2\;max}$) buckled in a global mode. Figure 12a illustrates the typical global and local collapse behaviors observed in the tubes.

Figure 12a presents the deformed geometry extracted from simulations, corresponding to the maximum and minimum root radii (R₂) for a grooved tube with three plies, a length (L) of 12R₁, and a distance parameter (λ/2) maintained at 1.5R₁. The deformed shape of the tubes indicates that those with the maximum root radius ($R_{2\;max}$) collapsed globally, a behavior that remained consistent even as the number of grooves (G) increased. This pattern was like that observed in tubes without grooves. Therefore, tubes with the maximum root radius ($R_{2\;max}$) exhibited a critical pressure very similar to that of tubes without grooves. In contrast, tubes featuring grooves with the minimum root radius ($R_{2\;min}$) collapsed locally and at a higher pressure, with geometric instability initiating in one of the subsections of the tube, as shown in Figure 12a. Furthermore, increasing the number of grooves resulted in shorter-length subsections, which collapsed at higher pressures in a three-lobed Mode III shape. The nomenclature for these angular mode shapes is based on the lobes observed in the deformed cross-section. For instance, a two-lobed deformed cross-section is labeled as Mode II collapse, while a three-lobed deformed cross-section is labeled as Mode III collapse. A detailed discussion of different mode shapes is provided by Matos et al. [16]. Figure 12b illustrates the Mode II and Mode III deformed shapes of the circular tube cross-section.

Figure 12c shows the normalized collapse pressure as a function of root radius for tubes with one, two, and three grooves. A higher number of grooves (or a smaller groove pitch distance) has a pronounced effect on increasing the collapse pressure of the tube. This suggests that the pitch distance of the grooves can be varied to achieve the desired collapse mode and collapse capacity.

**Figure 13:**
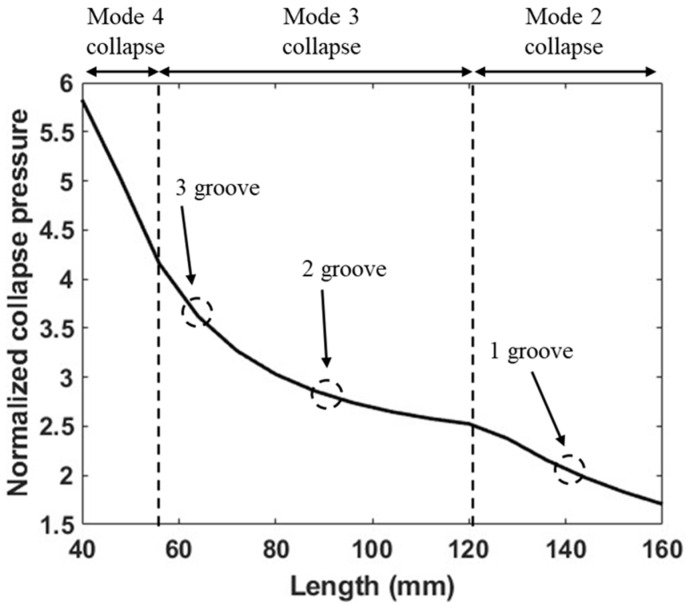
Normalized collapse pressure indicating change in collapse mode with change in tube length.

A change in the slope of collapse pressure increase is observed whenever a tube transitions to a higher collapse mode. Figure 13 illustrates this transition with changes in length for a tube with a D/t ratio of 30 (three-ply tubes with an internal radius of 25.4 mm). The subsection lengths corresponding to tubes of a length of $12R_{1}$ with one, two, and three grooves are marked in Figure 13, indicating their respective collapse modes. The collapse pressures were normalized with respect to the collapse pressure of a no-groove tube of length of $12R_{1}$ and with three plies.

##### Effect of Distance Parameter

**Figure 14:**
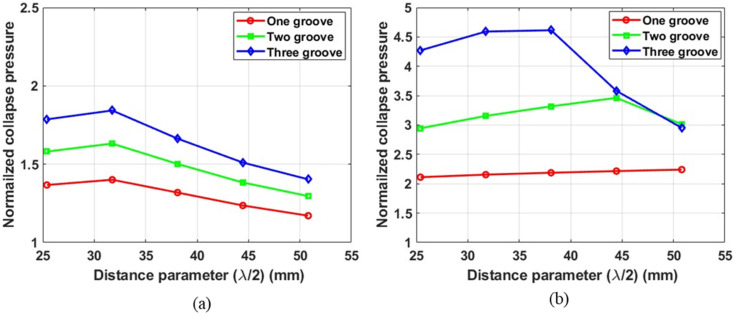
Normalized collapse pressure as a function of distance parameter for grooves of (**a**) intermediate root radius $\left(R_{2\;mid}\right)$ and (**b**) minimum root radius $\left(R_{2\;min}\right)$. Tube length = $8R_{1}$, No. of plies = 3.

The distance parameter (λ/2), defined in the earlier section, governs the steepness of the concave groove, as shown in Figure 8b. The parametric study results shown in Figure 9, Figure 10 and Figure 11 suggested a complex nonlinear dependence of the critical pressure of grooved tubes on the distance parameter. There was no clear trend observed in the collapse pressure for the variation in distance parameter. For shorter tubes with L = $8R_{1}$, the collapse pressure variation appeared unimodal. In general, the maximum collapse pressure occurred at an intermediate value of the distance parameter. The typical variation for shorter tubes is shown in Figure 14 for the intermediate as well as minimum root radius. With the decrease in the root radius of the tube, the maximum collapse pressure was attained at a greater magnitude of the distance parameter and vice versa.

**Figure 15:**
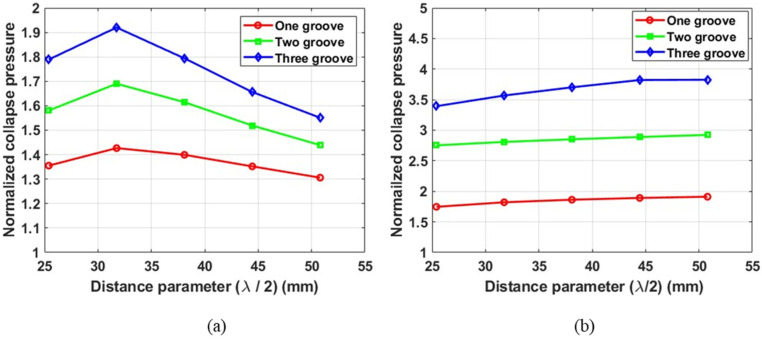
Normalized collapse pressure as a function of distance parameter for grooves of (**a**) intermediate root radius $\left(R_{2\;mid}\right)$ and (**b**) minimum root radius $\left(R_{2\;min}\right)$. Tube length = $12R_{1}$, No. of plies = 3.

However, the longer tubes showed a unimodal variation of collapse pressure with the distance parameter only for the intermediate root radius ($R_{2\;mid}$) geometry. The longer tubes with a minimum root radius ($R_{2\;min}$) exhibited a monotonically increasing trend in collapse pressure, albeit at a slow rate. Figure 15 illustrates the typical collapse pressure variation for a longer tube with L = $12R_{1}$ with an intermediate and minimum root radius.

**Figure 16:**
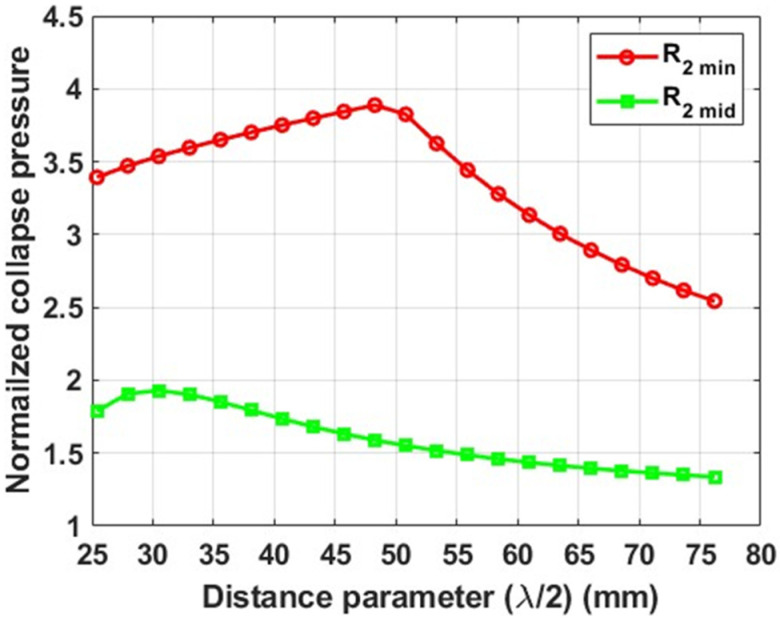
Typical normalized collapse pressure as function of λ/2, for tubes with grooves of minimum root radius $\left(R_{2\;min}\right)$ and grooves of intermediate root radius $\left(R_{2\;mid}\right)$. Tube length = $12R_{1}$, No. of plies = 3, No. of grooves = 3.

All tube configurations considered in the parametric study, except the longer tubes with a minimum root radius ($R_{2\;min}$), exhibit a unimodal trend of collapse pressure with respect to the distance parameter. Therefore, a shift in the maximum collapse pressure peak was anticipated to exist beyond the range of values of the distance parameter considered in the study. However, to confirm the anticipated trend, additional FE simulations were conducted, extending beyond the range of the parametric study, with typical results shown in Figure 16. The extended study results confirmed the anticipated trend, with tubes featuring both intermediate and minimum root radii exhibiting a unimodal trend in collapse pressure with the distance parameter.

**Figure 17:**
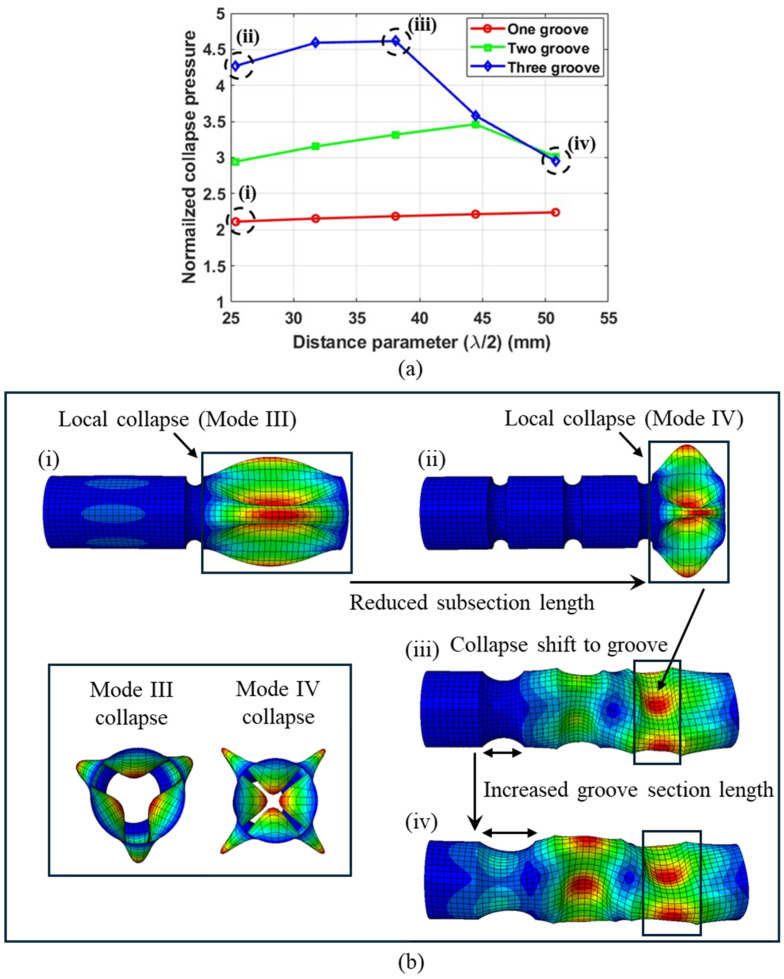
(**a**) Normalized collapse pressure as a function of λ/2. (**b**) Typical deformation behavior of tubes with grooves of minimum root radius. Tube length = $8R_{1}$, No. of plies = 3.

**Figure 18:**
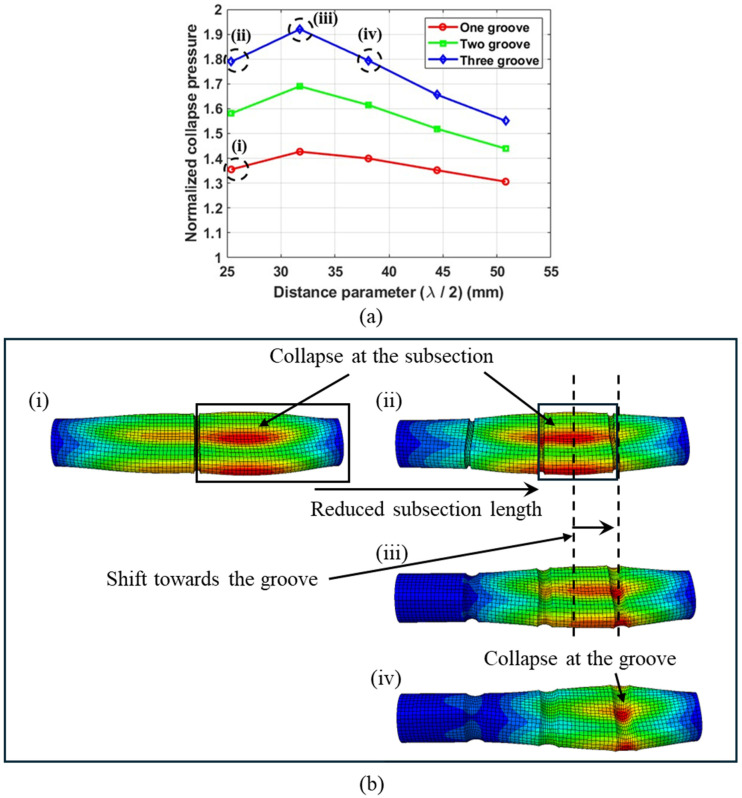
(**a**) Normalized collapse pressure as a function of λ/2. (**b**) Typical deformation behavior of tubes with grooves of intermediate root radius. Tube length = $12R_{1}$, No. of plies = 3.

This nonlinear variation in collapse pressure with the distance parameter results from the shift in the collapse initiation zone. To understand this behavior, the deformed geometries of the tubes were extracted from the FE simulation results. Typical results from tubes of two configurations were utilized to support this discussion, with their respective results presented in Figure 17 and Figure 18. Figure 17 illustrates the deformed shape of typical tubes with grooves of a minimum root radius $\left(R_{2min}\right),$ while Figure 18 shows the deformed shape of typical tubes with grooves of an intermediate root radius $\left(R_{2mid}\right)$.

Figure 17a illustrates the normalized collapse pressure as a function of the distance parameter for three-ply tubes with a length of $8\;R_{1}$ and grooves of a minimum root radius $\left(R_{2min}\right)$. The collapse pressures were normalized with respect to the collapse pressure of a no-groove tube of an identical geometry. Figure 17b illustrates the deformation behavior of tubes with four different grooves configurations, highlighting the unimodal collapse pressures trends.

Tubes with a maximum groove depth are divided into smaller subsections due to the high stiffness provided by the groove geometry. These tubes tend to collapse locally at the subsections. As the distance parameter (λ/2) increases, the length of the groove section also increases, which in turn reduces the length of the subsections, as shown in Figure 8b. This reduction in subsection length enhances their stiffness, resulting in an increase in collapse pressure as the distance parameter increases. However, the increased stiffness at the subsections comes at the expense of reduced stiffness at the groove section. Consequently, when the distance parameter reaches a certain threshold, the stiffness of the subsections exceeds that of the groove section, leading to collapse at the groove section. This point of collapse transition results in the highest collapse pressures as shown by (iii) in Figure 17a for tubes with three grooves. Once the tube transitions to collapse at the groove section, the collapse pressure is determined by the stiffness of the groove section. As the distance parameter increases, the length of the groove section also increases, as shown in Figure 17b(iii,iv). This results in a reduction in the stiffness of the groove section, leading to a decrease in collapse pressure.

This shift from collapse at the subsection to collapse at the groove section also depends on the subsection length. For shorter tubes ($8R_{1}$), the subsections are also shorter compared to the longer tubes ($12R_{1}$). Therefore, due to the higher stiffness of the subsections in shorter tubes, the transition from collapse at the subsections to collapse at the groove section occurs at a smaller value of the distance parameter.

The highest collapse pressures were observed in tubes with three grooves, followed by those with two grooves and one groove, respectively. This trend is attributed to the smaller subsections resulting from the increased number of grooves. Shorter tubes ($8R_{1}$) with one groove result in subsections of a length of 100 mm; this L/D ratio corresponds to Mode II collapse, as shown in Figure 13. However, shorter tubes ($8R_{1}$) with three grooves result in subsections of a length of 50 mm; this L/D ratio corresponds to Mode IV collapse, as shown in Figure 13. Therefore, smaller subsections collapse at higher modes, resulting in higher collapse pressures, as shown in Figure 17b(i,ii). This suggests that the pitch distance of the grooves can be varied to achieve the desired collapse mode and collapse capacity.

Figure 18a illustrates the normalized collapse pressure as a function of the distance parameter for three-ply tubes with a length of $12R_{1}$ and grooves of an intermediate root radius $\left(R_{2mid}\right)$. The collapse pressures were normalized with respect to the collapse pressure of a no-groove tube of an identical geometry. Figure 18b illustrates the deformation behavior of tubes with four different groove configurations, highlighting the unimodal collapse pressures trends.

The smallest value of the distance parameter (λ/2) results in the most abrupt change in groove geometry and the highest stiffness in the groove section. Due to the high stiffness of the groove section, collapse initiates at the subsection, as shown in Figure 18b(i,ii). As λ/2 increases, the groove steepness decreases, leading to a reduction in the stiffness of the groove section. However, an increase in λ/2  also shortens the length of the subsection, which in turn increases the stiffness of the subsection. Therefore, when the distance parameter reaches a certain threshold, the point of collapse initiation shifts toward the groove, as shown in Figure 18b(iii). This shift is attributed to the increased stiffness of the subsection and the reduced stiffness of the groove section. This shift results in the highest collapse pressures, as shown by (iii) in Figure 18a, for tubes with three grooves. Once collapse initiates at the groove section, a further increase in the λ/2 value leads to a decrease in collapse pressure. This decrease is attributed to the reduction in stiffness of the groove section, which results from the increased length of the groove section.

The highest collapse pressures were observed in tubes with three grooves, followed by those with two grooves and one groove, respectively. This is attributed to the smaller subsections resulting from the increased number of grooves, as shown in Figure 18b(i,ii). Table 6 summarizes the key findings from the parametric study.

## 4. Conclusions

An experimental study, followed by a parametric study, was conducted to investigate the effect of groove geometries on the collapse capacity of composite cylindrical tubes. An FE model was developed to simulate the hydrostatic loading of composite tubes, which was validated against experimental results. Once validated, the numerical model was used to perform the parametric study. Various groove configurations were explored for tubes of different geometries to assess their collapse behavior. The groove geometry was modified by adjusting the root radius and the distance parameter. The following conclusions were drawn from both the experimental and parametric studies:

1. The experimental results indicated a 20% increase in collapse pressures for tubes with a groove geometry;
2. The parametric study shows that groove geometries with a smaller root radius exhibit a higher stiffness. Tubes with grooves featuring a minimum root radius (*R*_2_*_min_*) collapsed locally at the subsections, resulting in higher collapse pressures. In contrast, tubes with grooves featuring a maximum root radius (*R*_2_*_max_*) collapsed globally, with collapse pressures like those of tubes without grooves;
3. In cases where the tubes collapsed locally, the collapse pressures showed dependence on the subsection geometry. Shorter subsection lengths resulted in higher-mode collapses, leading to an increase in collapse pressure;
4. A higher number of grooves leads to shorter subsection lengths. Shorter subsections exhibit greater stiffness, leading to higher-mode collapse and an increased collapse capacity. Consequently, tubes with three grooves demonstrated the highest collapse pressures. This suggests that the pitch distance of the grooves can be varied to achieve the desired collapse mode and collapse capacity;
5. The groove geometry with the smallest distance parameter (*λ*/2) results in the steepest groove, which exhibits the highest stiffness. An increase in the distance parameter leads to a decrease in groove stiffness and an increase in subsection stiffness. The highest collapse pressures were achieved at intermediate values of the distance parameter. The transition from collapse at the subsection to collapse at the groove section occurs at this intermediate value of the distance parameter, resulting in the highest collapse pressure. This indicates that, when designing tubes with grooves, this transitional value of the distance parameter must be considered;
6. The transitional value of the distance parameter depends on the following parameters:
  - Root radius of the groove: Grooves with a smaller root radius exhibit a higher stiffness. Therefore, tubes with smaller root radii will have a higher transition value of the distance parameter, and vice versa;
  - Tube length: Longer tubes correspond to longer subsections, which have a lower stiffness. Therefore, longer tubes will have a higher transition value of the distance parameter for the same groove geometry and vice versa;
  - Number of grooves: A higher number of grooves results in smaller subsections in tubes of the same length. Smaller subsections exhibit a higher stiffness. Therefore, tubes of the same length with a higher number of grooves will have a lower transition value of the distance parameter for the same groove geometry and vice versa.

## Figures and Tables

**Table 1:** Geometric details of composite tubes.

Type	L (mm)	$R_{1}$ (mm)	$R_{2}$ (mm)	λ/2 (mm)
No groove (NG)	330	25.4	-	-
With groove (WG)	330	25.4	23	48.5

**Table 2:** Mechanical properties of balanced twill-woven carbon fiber prepreg.

Mechanical Property	Values	Reference
$\mathrm{Elastic}\;\mathrm{Modulus},\;E_{11}$ and $E_{22}$ [GPa]	38.0 ± 0.5	ASTM D3039
$\mathrm{Poisson}’s\;\mathrm{Ratio},\;\nu_{12}$ and $\nu_{21}$	0.1	ASTM D3039
$\mathrm{Shear}\;\mathrm{Modulus},\;G_{12}$ [GPa]	3.0 ± 0.4	ASTM D5379
$\mathrm{Shear}\;\mathrm{Modulus},\;G_{13}$ and $G_{23}$ [GPa]	2.8 ± 0.2	ASTM D5379

**Table 3:** Summary of experimental results.

Specimen ID	Configuration	$P_{collapse}$ (MPa)
NG1	No groove	2.7
NG2	No groove	2.6
WG1	With groove	3.2
WG2	With groove	3.2

**Table 4:** Validation of FE model with experimental results.

Specimen ID	Configuration	$P_{collapse}$ (MPa) (Simulations)	$\mathrm{Avg}.\;P_{collapse}$ (MPa) (Experiments)	Error
NG	No groove	2.7	2.65	1.8%
WG	With groove	3	3.2	6%

**Table 5:** Summary of all parameters considered in parametric study.

Parameter	Variation
$\mathrm{Tube}\;\mathrm{radius}\;(R_{1})$ (mm)	25.4
Tube length (L) (mm)	$8R_{1},\;12R_{1},\;16R_{1},\;20R_{1}$
No. of plies (N)	2, 3, 4
$\mathrm{Root}\;\mathrm{radius}\;(R_{2})$ (mm)	$3\frac{R_{1}}{4},7\frac{R_{1}}{8},15\frac{R_{1}}{16}$
Distance parameter (λ/2) (mm)	$R_{1},\;1.25R_{1},\;1.5R_{1},\;1.75R_{1},\;2R_{1}$
No. of grooves (G)	1, 2, 3

**Table 6:** Summary of parametric study results.

Parameter	Effect
**Root radius** $\left(R_{2}\right)$	Grooves with a smaller root radius exhibit higher stiffness. Tubes with a minimum root radius (*R*_2_*_min_*) groove collapsed locally, resulting in higher collapse pressures.Tubes with a maximum root radius (*R*_2_*_max_*) groove collapsed globally, with collapsepressures similar to those of no-groove tubes.
**Tube length** (L)	Shorter tubes exhibited a greater increase in collapse pressures compared to longer tubes with the same number of grooves. Shorter tubes with grooves have shorter subsections, which result in higher-mode collapses and higher collapse pressures.
**No. of grooves** (G)	A higher number of grooves results in shorter subsections, which exhibit a higher stiffness and greater collapse capacity. Tubes with three grooves demonstrated the higher collapse pressures.
**Distance parameter** (λ/2)	Grooves with the smallest distance parameter produce the steepest groove with the highest stiffness. Tubes with grooves exhibited the greatest increase in collapse pressures at intermediate values of the distance parameter. The transition value of the distance parameter that results in the highest collapse pressures depends on the following factors: (a) Root radius: Grooves with a smaller root radius result in a higher transition value of *λ*/2; (b) Tube length: Longer tubes exhibit a higher transition value for *λ*/2; (c) No. of grooves: Tubes with more grooves have smaller subsections with a higher stiffness, leading to a lower transition value of *λ*/2.

## Data Availability

The original contributions presented in this study are included in the article. Further inquiries can be directed to the corresponding author.
